# Examining the Genetic and Environmental Associations between Autistic Social and Communication Deficits and Psychopathic Callous-Unemotional Traits

**DOI:** 10.1371/journal.pone.0134331

**Published:** 2015-09-01

**Authors:** Elizabeth O’Nions, Beata Tick, Fruhling Rijsdijk, Francesca Happé, Robert Plomin, Angelica Ronald, Essi Viding

**Affiliations:** 1 MRC Social, Genetic and Developmental Psychiatry Centre, Institute of Psychiatry, Psychology and Neuroscience, King’s College London, London, United Kingdom; 2 Developmental Risk & Resilience Unit, Clinical, Educational, and Health Psychology Research Department, Division of Psychology and Language Sciences, University College London, London, United Kingdom; 3 Department of Psychological Sciences, Birkbeck, University of London, London, United Kingdom; University of London, UNITED KINGDOM

## Abstract

**Background:**

Difficulties in appropriate social interaction are characteristic of both children with autism spectrum disorders and children with callous-unemotional traits (who are at risk of developing psychopathy). Extant experimental studies suggest that the nature of atypical social cognition that characterises these two profiles is not identical. However, ‘empathizing’ difficulties have been hypothesised for both groups, raising questions about the degree of aetiological separation between social impairments that characterize each disorder. This study explored the relative contribution of independent vs. shared aetiological influences to social and communication impairments associated with autistic traits and callous-unemotional traits, indexed by parent-report in a population-based cohort of twins.

**Methods:**

Participants were over 5,000 twin pairs from a UK cohort (the Twins Early Development Study; TEDS), assessed for callous-unemotional traits at 7 years and autistic social and communication impairments at 8 years. Multivariate model-fitting was used to explore the relative contribution of independent vs. overlapping genetic/environmental influences on these traits.

**Results:**

Both social and communication impairments and callous-unemotional traits were highly heritable, although the genetic and environmental influences accounting for individual differences on each domain were predominantly independent.

**Conclusions:**

Extant evidence from experimental and neuro-imaging studies has suggested that, despite some superficially overlapping behaviours, the social difficulties seen in children with autism spectrum disorders and callous-unemotional traits are largely distinct. The current study is the first to demonstrate considerable aetiological independence of the social interaction difficulties seen in children with autism spectrum disorders and those with callous-unemotional traits.

## Introduction

Individuals who have either high levels of autism spectrum disorder (ASD) or callous-unemotional traits often display atypical patterns of social interaction. Both groups can, on the surface, behave in ways that lack sensitivity or empathy for other people and their needs. Social and communication difficulties that form part of the diagnostic criteria for ASD reflect disturbances in the ability to relate to others and understand their thoughts and intentions, with consequent problems with friendships, play, and initiating and responding to social overtures [1]. Callous-unemotional traits reflect disturbances in the ability to empathise and affiliate with others [2]. These traits can be detected in developmental samples and significantly increase risk for psychopathy in adulthood [3]. The term ‘empathizing impairment’ has been used to cover both patterns of difficulty [4].

A number of past studies have used questionnaire measures and found that both individuals with ASD or callous-unemotional traits display difficulties in empathic and pro-social behaviour [5, 6]. Data from cognitive neuroscience allows for more precise assessment of different processes that might explain difficulties in empathic and pro-social behaviour in those with ASD or callous-unemotional traits. These studies suggest that ASD and callous-unemotional traits are characterised by at least partially distinct impairments in social awareness and empathy. Children with ASD have difficulty intuiting others’ thoughts, beliefs and intentions [7]. However, they appear to show intact responses to others’ distress, suggesting that basic reactivity to emotions is preserved [8, 9]. As such, abnormal social responses in ASD, including behaviours that on the surface appear un-empathic, may stem from deficits in identifying others’ inner states, rather than basic reactivity to emotional stimuli. In contrast, children with callous-unemotional traits appear to have good insight into others’ mental states (as measured by Theory of Mind tasks) but lack concern for others’ feelings (i.e. “knowing” but not “caring”) [10, 11]. It is thus plausible that although both ASD and callous-unemotional traits are negatively associated with general measures of empathic and pro-social behaviours, these associations may be driven by different underlying aetiological and socio-affective vulnerabilities.

Although there has been some progress in comparing and contrasting the socio-affective profile of individuals with ASD and callous-unemotional traits [10, 12, 13], we still know remarkably little about the degree to which these features are independent vs. co-occur in the general population and the degree to which they have independent vs. shared genetic and environmental influences. Both ASD traits and callous-unemotional traits are continuously distributed, with individuals at the extreme representing disordered functioning in each case [13–16]. No studies to date have investigated the phenotypic overlap between the socio-affective behaviours of ASD and callous-unemotional traits in the general population. Previous findings indicate that autistic and callous-unemotional traits are each strongly heritable, with non-shared environmental influences accounting for the less than perfect concordance between identical twins [17–20]. One previous study [21] explored the extent to which autistic and psychopathic traits are aetiologically independent in 642 twin pairs aged 8–10 years, focusing on the broad constructs of ASD and psychopathy, rather than aspects most closely related to un-empathic behaviours (i.e. social aspects of the autistic phenotype and callous-unemotional traits). That study reported a large degree of aetiological independence between the constructs, though this may have been partly influenced by low covariance between conceptually distant components of the phenotypes (e.g. narcissism / impulsivity in psychopathy and rigid and repetitive behaviours in ASD). To date, no study has explored whether this pattern holds for the traits capturing atypical socio-affective features in particular (i.e. social interaction, social communication, and callous-unemotional traits); this was the purpose of the present study. The present study thus aimed to determine the extent to which these traits are driven by distinct vs. overlapping aetiological influences in a general population sample of twins aged 7–8 years.

## Methods

### Participants

Participants were drawn from the UK Twins Early Development Study (TEDS), a population-based cohort of twins born in England and Wales between 1994 and 1996 [22]. The Twins Early Development Study cohort was established through birth records, and the sample at age 7–8 is considered representative of the UK population [23]. Zygosity was assessed using a standard zygosity questionnaire, which proved to be accurate in 95% of the cases [24]. For the remaining pairs, zygosity was assessed via full DNA tests. The Twins Early Development Study was approved by the King’s College London research ethics committee and all participants gave written informed consent after a complete description of the study.

The sample for parent-rated callous-unemotional traits consisted of 7278 pairs (2591 monozygotic (MZ), 2386 dizygotic same-sex (DZ-SS) and 2301 dizygotic opposite-sex twin pairs (DZ-OS)). For social interaction and social communication subscales (derived from the Childhood Autism Spectrum Test) [25], 6402 twin pairs were included (MZ N = 2253; DZ SS N = 2090; DZ OS N = 2059). Overall, there were 5630 pairs for whom ratings were available for callous-unemotional traits, as well as the social interaction and social communication traits. Exclusion criteria were significant pre- or peri-natal complications or a syndromic/chromosomal disorder other than ASD (e.g. Down’s Syndrome), or if zygosity could not be established. Mean age of twins was 7.1 years (SD = .25) when rated by parents on callous-unemotional traits and 7.9 years (SD = .53) when parents rated social interaction and social communication traits.

### Measures

Callous-unemotional traits were rated by parents. The callous-unemotional traits measure consisted of the four questions incorporated in the DSM-5 specifier (originally from the Antisocial Process Screening Device [26], a well validated measure of psychopathic tendencies in adolescents) and four additional items relevant to callous-unemotional traits from the Strengths and Difficulties Questionnaire [27, 28] (8 items; S1 Table). Items were rated on a three-point Likert scale (0 = not true, 1 = somewhat true, 2 = certainly true). Scores ranged from 0–16 points. Mean scores were calculated for each twin, based on at least 4 item scores (any twin with fewer values than 4 scores was excluded from analysis). Assessment of callous-unemotional traits using this method resulted in a modest internal consistency (Cronbach’s Alpha α = .47).

Autistic traits were assessed by parents using the Childhood Autism Spectrum Test [25]. This measure was designed to indicate the presence of autistic traits in a non-clinical setting and has good reliability and internal consistency. Subscales have been identified within the measure based on the DSM-IV criteria for social interaction (12 items) and social communication (12 items) difficulties, plus rigid and repetitive behaviours (7 items). Social interaction and social communication subscales were used in the current study (S1 Table) [29]. In line with previous studies [29–31], these two subscales were kept separate. Items were rated on a two-point Likert scale (0 = No; 1 = Yes). Subscale scores were calculated on the basis of at least six item scores. The social interaction and social communication scales showed a modest internal consistency (α = .56 and α = .66 respectively), similar to previous reports [29–30]. The response rate for the booklet containing the measure of callous-unemotional traits was 54.2%. For the booklet containing the social interaction and communication scales, the response rate was 48.7%. The TEDS sample has been shown to be representative of the UK general population on a range of indicators, including maternal educational level [32] Participation rates are complex in a 15-year longitudinal study that does not aim to create an epidemiological sample, but rather a representative sample of twins, and thus does not push families to participate at each wave of assessment.

Teacher ratings were also available for callous-unemotional traits only, though these were not the primary focus of this study. Model fitting results presented in the main body of the text refer to models that included parent data only to avoid an artificial reduction in the possibility of identifying shared aetiological effects due to reduced rater agreement. However, results from models using teacher-reported callous-unemotional traits and parent-reported social interaction and social communication are included in S1 Analysis, S2 Fig and S3 and S4 Tables.

### Data analyses

The current study employed the classic twin design to partition the variance and covariance within traits into unique and common genetic and environmental influences, by comparing the phenotypic resemblance separately for monozygotic and dizygotic twins within as well as across traits. The genetic relatedness of monozygotic twins was set to a coefficient of 1.0 and for dizygotic twins 0.5 (mirroring 100% and on average 50% of segregating alleles shared between the two types of twins). The twin model assumes that twin similarity is due to additive genetic and shared environmental factors. MZ dissimilarity can only be due to non-shared environmental influences, which also includes measurement error [33, 34]. All scores were controlled for the effects of age and sex [35]. Analyses used full information maximum likelihood on twin data in the Structural Equation Model program OpenMx [36] a matrix optimization package in the R environment (www.R-project.org) [37]. The goodness of fit of a particular model was indicated by a fit statistic of minus twice the log likelihood. The goodness of fit of alternative sub-models was established on the basis of differences in likelihood, which is distributed as a chi-square (χ^2^) statistic. A significant χ^2^ of a sub-model indicated that it fitted the data worse than the full model.

### Constrained Correlation Model

A Constrained Correlation Model was fitted for the 5 sex-by-zygosity groups, providing summary statistics reflecting the importance of genetic factors on variation across each trait and the covariance between the traits. The ratio between monozygotic and dizygotic cross-twin cross-trait correlations for the three traits provides an indication of the relative contributions of *common* latent additive genetic, shared environmental and non-shared environmental factors. The estimates of monozygotic and dizygotic cross-twin within-trait correlations estimate the univariate genetic and environmental influences on the three traits separately. This model also allows the inspection of possible correlation ratio differences for monozygotic and dizygotic twins of different sexes, thus indicating whether it would be of interest to examine sex-differences formally.

### Multivariate model fitting—Independent Pathway Model

Our primary question of interest was the magnitude of independent vs. overlapping genetic and environmental variance acting on the three traits. An independent pathway model was used to estimate these [38]. In this model, the covariance between the three traits is partitioned into common genetic, common shared environmental and common non-shared environmental factors that overall reflect the aetiological co-occurrence across the traits. Non-overlapping genetic and environmental influences are captured in the form of unique genetic, unique shared and unique non-shared environmental influences acting on each trait separately. These account for the remainder of variance not explained by the common latent genetic and environmental paths. In addition, we fitted a Cholesky model [39] to estimate the genetic and environmental correlations between callous-unemotional traits, social interaction, and social communication variables, to examine the aetiological overlap between each pair of variables in turn.

## Results

There was a modest but significant phenotypic overlap between parent ratings for the three traits of interest (social interaction and social communication, r = .27; p<.001, callous-unemotional traits and social interaction: r = .22, p<.001, and callous-unemotional traits and social communication: r = .21, p<.001). Table 1 illustrates cross-twin within-trait (shown on the diagonal) and cross-twin cross-trait correlations (shown on the off-diagonal) for participants grouped by zygosity and gender. The monozygotic and dizygotic twin correlations (across both males and females) on the diagonal show a 2:1 ratio, meaning that all three traits are significantly influenced by additive genetic factors (A) with negligible input of shared environmental factors (C) and with remaining variance explained by non-shared environmental factors (E). The higher MZ than DZ cross-twin cross-trait correlations on the off-diagonal suggest some common genetic influences between callous-unemotional traits, social interaction and social communication, with a negligible amount of covariance explained by shared environmental factors.

**Table 1:** Cross-twin within-trait and cross-trait correlations, stratified by zygosity and gender. Cross-trait correlations are below the diagonals; within-trait correlations are on the diagonals. Abbreviations: CU = callous-unemotional; SI = Social interaction; SC = Social communication.

Monozygotic Males	CU, Twin 1	SI, Twin 1	SC, Twin 1
CU, Twin 2	.68 (.65 - .70)	-	-
SI, Twin 2	.24 (.20 - .27)	.76 (.73 - .78)	-
SC, Twin 2	.23 (.20 - .27)	.26 (.23 - .29)	.81 (.79 - .82)
Dizygotic Males	CU, Twin 1	SI, Twin 1	SC, Twin 1
CU, Twin 2	.34 (.29 - .39)	-	-
SI, Twin 2	.07 (.03 - .11)	.30 (.24 - .35)	-
SC, Twin 2	.10 (.06 - .15)	.06 (.01 - .10)	.41 (.35 - .45)
Monozygotic Females	CU, Twin 1	SI, Twin 1	SC, Twin 1
CU, Twin 2	.64 (.61 - .67)	-	-
SI, Twin 2	.17 (.14 - .21)	.70 (.68 - .73)	-
SC, Twin 2	.16 (.13 - .20)	.18 (.15 - .22)	.80 (.78 - .82)
Dizygotic Females	CU, Twin 1	SI, Twin 1	SC, Twin 1
CU, Twin 2	.31 (.27 - .35)	-	-
SI, Twin 2	.07 (.03 - .12)	.34 (.29 - .39)	-
SC, Twin 2	.12 (.08 - .16)	.10 (.06 - .14)	.50 (.46 - .54)
Dizygotic opposite-sex	CU, Twin 1	SI, Twin 1	SC, Twin 1
CU, Twin 2	.44 (.39 - .48)	-	-
SI, Twin 2	.09 (.05 - .13)	.29 (.23 - .34)	-
SC, Twin 2	.13 (.09 - .16)	.11 (.07 - .15)	.46 (.41 - .50)

Key points
- Difficulties in appropriate social interaction are characteristic of both children with autism spectrum disorders and children with callous-unemotional traits. - Experimental studies suggest that the nature of atypical social cognition that characterises these two profiles is not identical. However, ‘empathizing’ difficulties have been reported in both groups. - Our findings indicate that both social and communication impairments and callous-unemotional traits show modest phenotypic overlap. Aetiological influences accounting for individual differences on each domain were predominantly independent. - Although both children with high levels of ASD traits and children with high levels of callous-unemotional traits exhibit difficulties in appropriate social behaviour, the underlying drivers of these impairments are predominantly distinct.

The constrained-correlation model indicated that within-trait and cross-trait correlation values for monozygotic and dizygotic twins were similar across males and females; meaning that the magnitude of common and unique genetic and environmental influences is similar across genders. Comparison of correlations between same-sex and opposite-sex dizygotic twin pairs indicated similar degrees of resemblance, suggesting that the same genetic factors influence variation in both males and females. As such, dizygotic opposite-sex pairs were retained in the multivariate analysis.

### Independent Pathway Model

**Fig 1:**
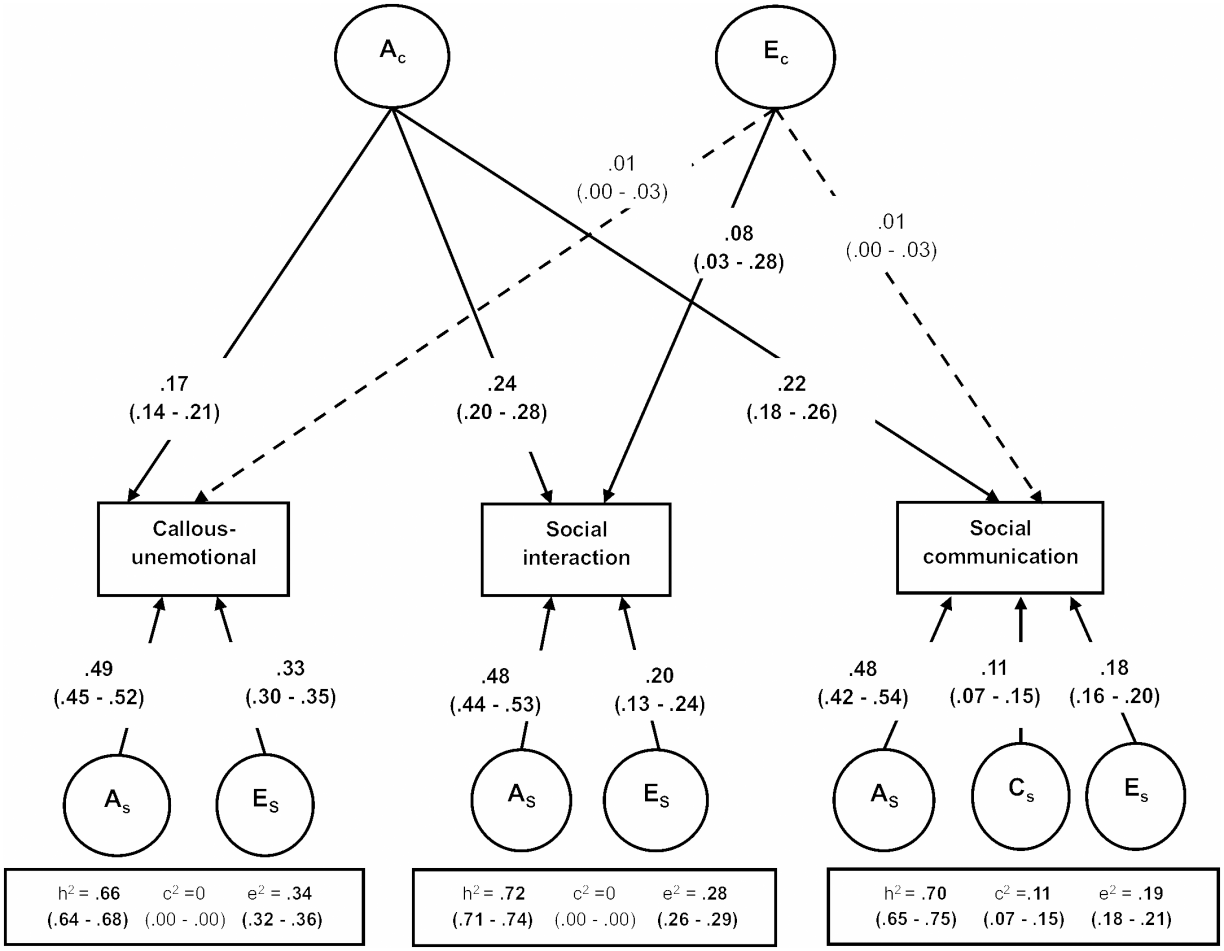
Path diagram of the reduced Independent Pathway model. Paths estimates are expressed as the proportion of variance accounted for by common or specific aetiological factors. For callous-unemotional, 26% of overall genetic influences are due to shared factors and 74% due to trait specific factors. For social interaction, 33% of overall genetic influences are due to shared factors and 67% due to trait specific factors. For social communication, 31% of overall genetic influences are due to shared factors and 69% due to trait specific factors. Dotted arrows indicate a non-significant path estimate. A_c_ = common genetic factors; E_c_ = common non shared environmental factors; A_s_ = specific genetic factors; C_s_ = specific shared environmental factors; E_s_ = specific non shared environmental factors. h^2^ = proportion of variance on the trait explained by additive genetic influences; c^2^ = proportion of variance explained by shared environmental influences; e^2^ = proportion of variance explained by non-shared environmental influences.

Results of the multivariate model-fitting analyses are shown in S2 Table. In the full model, parameter estimates for several unique and common shared and non-shared environmental paths were equal to 0, suggesting a more parsimonious model might provide a better fit to the data. We fitted 5 different nested sub-models, dropping model paths related to both unique and common shared and non-shared environmental parameters (S2 Table). The best fitting model is shown in Fig 1, and from this we derive our estimates of the relative importance of independent and common aetiological influences.

For callous-unemotional, social interaction and social communication difficulties, most of the overall genetic influences on population variance were due to variable-specific genetic factors, explaining 74%, 67% and 69% of the heritability, respectively. The common genetic factor acting on all three traits explained a smaller proportion of the heritability: 26%, 33% and 31%, respectively. Specific shared environments were significant only for social communication, albeit small in magnitude, with no common shared environmental influences acting on the covariance between the three variables. Trait-specific non-shared environmental influences were significant for all variables, whereas social interaction appeared to be influenced by non-shared environmental influences that stemmed from the common non-shared environments (E) path, although this is effectively operating as trait-specific non-shared environmental effect as it did not contribute to variance on callous-unemotional traits or social interaction.

Given the possibility that pleiotropic effects of genes may act on a pair of variables, but not on all three (as assessed by the independent pathway model), we also derived a correlated factors solution of the Cholesky model [38]. This model produces estimates of genetic (r_A_) and environmental (r_C_ and r_E_) correlations between the variables, irrespective of the magnitude of these factors acting on the variable alone (results presented in S1 Fig). For parent-report data, a modest genetic correlation was reported between callous-unemotional traits and social interaction (r_A_ = .31) and between callous-unemotional traits and social communication (r_A_ = .23). Low non-shared environmental correlations were also observed between callous-unemotional traits and social interaction (r_E_ = .10) and between callous-unemotional traits and social communication (r_E_ = .07). The genetic and environmental overlap between social interaction and social communication difficulties was also modest (r_A_ = .32; r_E_ = .14) as reported previously [29].

The supplemental analysis using teacher-rated callous-unemotional traits and parent rated autistic traits (see S1 Analysis) to fit an independent pathway model suggested a similar pattern of results, with a slightly smaller proportion of genetically driven variance on the traits related to common latent genetic influences (15%, 26% and 39%, for callous-unemotional, social interaction and social communication respectively), as might be expected given the smaller phenotypic correlations between parent and teacher ratings (S2 Fig and S3 and S4 Tables). This provides further validation for largely independent aetiological effects on callous-unemotional traits and social interaction and social communication difficulties associated with ASD.

## Discussion

A large body of clinical research demonstrates that difficulties in appropriate empathic and pro-social interaction characterize both children with ASD and children with callous-unemotional traits [5, 6]. There is also evidence that at least partially distinct impairments in theory of mind and empathy underlie ASD and callous-unemotional traits [10, 12, 13]. It is thus plausible that although both ASD and callous-unemotional traits are negatively associated with general measures of empathic and pro-social behaviours, these associations may be driven by different underlying aetiological and socio-affective vulnerabilities.

This study was the first to examine systematically the degree of overlap between behavioural measures of socio-affective difficulties in individuals with high levels of autistic or callous-unemotional traits in a general population sample of twins aged 7–8 years. The findings show a modest degree of phenotypic correlation between the traits, suggesting that, in the general population, these traits are predominantly distinct. Because we studied twins, we were also able to examine the aetiological overlap between social interaction and social communication difficulties associated with ASD, and callous-unemotional traits (i.e. the degree to which independent vs. overlapping genetic or environmental influences are important in the development of individual differences in these traits). Twin model-fitting indicated that social interaction, social communication, and callous-unemotional traits are all strongly heritable, with negligible impact of shared environmental effects, as reported previously. Less than perfect correlations between identical twins also indicate that child-specific environmental factors are important in explaining variation on these three traits [17, 27, 29, 40].

The independent pathway model indicated that aetiological influences on the three traits are mostly unique to each construct. Independent genetic influences explained 74% of total genetic variance for callous-unemotional; 67% of total genetic variance for social interaction; and 69% for social communication. The remainder of the genetic variance was explained by genetic factors shared between the three traits.

These findings are consistent with previous reports of predominantly distinct genetic influences on autistic and psychopathic traits (including conceptually distant components such as narcissism in psychopathy and rigid repetitive behaviours in ASD) [21]. Our findings show that genetic separation holds when focusing on the sub-components specifically related to social interaction. The present findings suggest that mostly independent genetic vulnerabilities may drive the distinct neuro-cognitive atypicalities that predispose to autistic or callous-unemotional traits respectively, and indicate that within a broad category of so-called ‘empathizing’ impairments, predominantly different aetiological influences are at play.

Overlapping genetic influences accounted for a modest amount of the total genetic influences acting on the three traits (callous-unemotional: 26%, social interaction: 33% and social communication: 31%), also reflected by the modest genetic correlation values. The modelling results indicate that the modest degree of phenotypic overlap observed between the three traits is largely driven by shared genetic, rather than environmental factors. This suggests that there may be some shared processes (or a third trait dimension, for example alexithymia [41] or externalising problems [42, 43]), which co-occur in both conditions and contribute to difficulties evident in each. More work is needed to investigate possible shared cognitive processes, and explore why some individuals may exhibit a ‘double hit’ of difficulties associated with both ASD and CU traits [44].

A second possible interpretation is that the observed modest genetic overlap is due to genetic liability to one phenotype (e.g. social communication difficulties) increasing risk for high ratings on the other phenotype (e.g. callous-unemotional behaviour); for example, children with poor communication skills may show atypical expressions of empathy. As such, the apparent aetiological overlap between the phenotypes may be a consequence of the observer-questionnaire based approach to measuring the constructs. Future studies using experimental measures of cognitive and affective empathy in children with these profiles would be helpful in determining the nature of the apparent overlap reported here.

A strength of this study is that our sample is a general population cohort, allowing us to explore aetiological overlap between the traits across the entire distribution of severity, rather than just within a clinical sample. Limitations of the present study include the relatively low alpha for the parent-rated callous-unemotional traits measure, which could have limited the ability to detect genetic influences, resulting in inflated estimates of error. However, our scale properties are consistent with previous reports of low alphas in the assessment of callous-unemotional traits using items from the anti-social process screening device [45]. Notably, the analysis conducted using teacher-rated callous-unemotional traits produced similar estimates of heritability (66% using parent-report and 71% using teacher-report), despite the teacher-rated callous-unemotional trait measure having a substantially higher alpha (.75). Regardless, a replication with ASD and CU measures with stronger internal consistency, as well as collection of multi-informant data would provide further confidence in these findings. An additional limitation was that the data on callous-unemotional traits was collected when children were on average ten months younger than when the data on ASD traits was collected. This may have reduced the size of the observed association between callous-unemotional and ASD traits and future studies with both measures obtained at a single time point will be helpful.

## Conclusion

Overall, the current study yielded several important findings. Firstly, the correlation between social, communication and callous-unemotional traits was modest across this population sample, indicating that these traits can be disentangled phenotypically in a 7–8 year-old cohort of twins. Predominantly trait-specific genetic and environmental influences contribute to social interaction difficulties, social communication difficulties and callous-unemotional traits. This finding is consistent with behavioural and neuro-imaging studies that report separate cognitive and neural correlates of these profiles in affected groups and in population samples with high levels of these traits [10, 12, 13]. The small but significant degree of phenotypic co-variation between the three traits was predominantly driven by genetic influences that impacted all three.

The implications of these findings are that although both children with high levels of ASD traits and children with high levels of callous-unemotional traits exhibit difficulties in appropriate social behaviour, the underlying drivers of these impairments appear to differ between the profiles. Rating scales for ASD and CU traits appear to differentiate appropriately distinct social/ behavioural constructs that are largely unrelated aetiologically. The small degree of aetiological overlap observed here be a consequence of the questionnaire based approach to measuring the constructs used here, or could reflect a shared third trait that impacts both profiles, such as alexithymia. Further research is needed to examine and compare the neuro-cognitive basis of difficult or un-empathic behaviour seen in children with ASD traits or callous-unemotional traits.

## Supporting Information

S1 Analysis Teacher-reported callous-unemotional traits and parent-reported social interaction and social communication difficulties. (DOCX)

S1 Fig Cholesky ACE model: Correlated Factors Solution outputs using parent report data on callous-unemotional traits, social interaction and social communication. Abbreviations: A_1-3_ = Additive genetic factors modelled for each trait separately; C_1&3_ = Shared environmental factors modelled for each trait separately; E_1-3_ = Non-shared environmental factors modelled for each trait separately. Correlations for A, C and E factors between pairs of traits are shown. Paths from the A, C and E factors on to the traits reflect the estimated role of each of these factors in contributing to variance on the trait. Path C_2_ was removed as common environmental factors did not contribute significantly to variance on social interaction difficulties.
(PDF)

S2 Fig Path diagram of the reduced Independent Pathway model. Paths estimates are expressed as the proportion of variance accounted for by common or specific aetiological factors. For callous-unemotional, 15% of overall genetic influences are due to shared factors and 85% due to trait specific factors. For social interaction, 26% of overall genetic influences are due to shared factors and 74% due to trait specific factors. For social communication, 39% of overall genetic influences are due to shared factors and 61% due to trait specific factors. Dotted arrows indicate a non-significant path estimate. A_c_ = common genetic factors; E_c_ = common non shared environmental factors; A_s_ = specific genetic factors; C_s_ = specific shared environmental factors; E_s_ = specific non shared environmental factors. h^2^ = proportion of variance on the trait explained by additive genetic influences; c^2^ = proportion of variance explained by shared environmental influences; e^2^ = proportion of variance explained by non-shared environmental influences.
(PDF)

S1 Table Items measuring callous-unemotional, social interaction and social communication traits. (DOCX)

S2 Table Model fitting results for the Independent Pathway Models using parent-report data for callous-unemotional traits, social interaction and social communication difficulties. (DOCX)

S3 Table Cross-twin within-trait and cross-trait correlations, stratified by zygosity and gender using teacher-report data for callous-unemotional traits, and parent-report data for social interaction and social communication difficulties. (DOCX)

S4 Table Model fitting results for the Independent Pathway Models using teacher-report data for callous-unemotional traits, and parent-report data for social interaction and social communication. (DOCX)

## References

[1] Wing L, Gould J. Severe impairments of social interaction and associated abnormalities in children: epidemiology and classification. Journal of Autism and Developmental Disorders, 1979 9: p. 11–29. 155684 10.1007/BF01531288

[2] Frick PJ, O’Brien BS, Wooton JM, McBurnett K. Psychopathy and conduct problems in children. Journal of Abnormal Psychology, 1994 103: p. 700–707. 7822571 10.1037//0021-843x.103.4.700

[3] Viding E, Fontaine NM, McCrory EJ. Antisocial behaviour in children with and without callous-unemotional traits. Journal of the Royal Society of Medicine, 2012 105: p. 195–200. 10.1258/jrsm.2011.110223 22637770 PMC3360537

[4] Baron-Cohen S. Zero Degrees of Empathy: A New Theory of Human Cruelty. Penguin/Allen Lane. 2011.

[5] Dadds MR, Hawes DJ, Frost AD, Vassallo S, Bunn P, Hunter K, et al Learning to 'talk the talk': The relationship of psychopathic traits to deficits in empathy across childhood. Journal of Child Psychology and Psychiatry and Allied Disciplines, 2009 50: p. 599–606. 10.1111/j.1469-7610.2008.02058.x 19445007

[6] Auyeung B, Wheelwright S, Allison C, Atkinson M, Samarawickrema N, Baron-Cohen S. The children's empathy quotient and systemizing quotient: Sex differences in typical development and in autism spectrum conditions. Journal of Autism and Developmental Disorders, 2009 39: p. 1509–1521. 10.1007/s10803-009-0772-x 19533317

[7] Baron-Cohen S. Are autistic children "Behaviorists"? An examination of their mental-physical and appearance-reality distinctions. Journal of Autism and Developmental Disorders, 1989 19: p. 579–600. 2606886 10.1007/BF02212859

[8] Blair RJR. Psychophysiological responsiveness to the distress ofothers in children with autism. Personality and Individual Differences, 1999 26(3): p. 477–485.

[9] Dziobek I, Rogers K, Fleck S, Bahnemann M, Heekeren HR, Wold OT, et al Dissociation of cognitive and emotional empathy in adults with Asperger syndrome using the Multifaceted Empathy Test (MET). Journal of Autism and Developmental Disorders, 2008 38: p. 464–473. 17990089 10.1007/s10803-007-0486-x

[10] Jones AP, Happé FG, Gilbert F, Burnett S, Viding E. Feeling, caring, knowing: different types of empathy deficit in boys with psychopathic tendencies and autism spectrum disorder. Journal of Child Psychology and Psychiatry, and Allied Disciplines, 2010 51: p. 1188–1197. 10.1111/j.1469-7610.2010.02280.x 20633070 PMC3494975

[11] Schwenck C, Mergenthaler J, Keller K, Salehi S, Taurines R, Romanos M, et al Empathy in children with autism and conduct disorder: Group-specific profiles and developmental aspects. Journal of Child Psychology and Psychiatry and Allied Disciplines, 2012 53: p. 651–659. 10.1111/j.1469-7610.2011.02499.x 22118246

[12] O'Nions E, Sebastian CL, McCrory E, Chantiluke K, Happé F, Viding E. Neural bases of Theory of Mind in children with autism spectrum disorders and children with conduct problems and callous-unemotional traits. Developmental Science, 2014 17(5): p. 786–796. 10.1111/desc.12167 24636205 PMC4316185

[13] Lockwood PL, Bird G, Bridge M, Viding E. Dissecting empathy: high levels of psychopathic and autistic traits are characterized by difficulties in different social information processing domains. Frontiers in Human Neuroscience, 2013 7: p. 760–760. 10.3389/fnhum.2013.00760 24294197 PMC3826592

[14] Constantino JN, Przybeck T, Friesen D, Todd RD. Reciprocal social behavior in children with and without pervasive developmental disorders. Journal of Developmental & Behavioral Pediatrics, 2000 21: p. 2–11. 10706343 10.1097/00004703-200002000-00002

[15] Murrie DC, Marcus DK, Douglas KS, Lee Z, Salekin RT, Vincent G. Youth with psychopathy features are not a discrete class: A taxometric analysis. Journal of Child Psychology and Psychiatry and Allied Disciplines, 2007 48: p. 714–723. 10.1111/j.1469-7610.2007.01734.x 17593152

[16] Wallace GL, Shaw P, Lee NR, Clasen LS, Raznahan A, Lenroot RK, et al Distinct Cortical Correlates of Autistic versus Antisocial Traits in a Longitudinal Sample of Typically Developing Youth. Journal of Neuroscience, 2012 p. 4856–4860. 10.1523/JNEUROSCI.6214-11.2012 22492041 PMC3342014

[17] Viding E, Frick PJ, Plomin R. Aetiology of the relationship between callous-unemotional traits and conduct problems in childhood. British Journal of Psychiatry, 2007 p.190. 10.1192/bjp.190.5.s33 17470941

[18] Ronald A, Happé F, Price TS, Baron-Cohen S, Plomin R. Phenotypic and genetic overlap between autistic traits at the extremes of the general population. Journal of the American Academy of Child and Adolescent Psychiatry, 2006 45(10): p. 1206–1214. 17003666 10.1097/01.chi.0000230165.54117.41

[19] Hoekstra RA, Bartels M, Verweij CJ, Boomsma DI. Heritability of autistic traits in the general population. Archives of Pediatrics & Adolescent Medicine, 2007 161(4): p. 372–377. 17404134 10.1001/archpedi.161.4.372

[20] Larsson H, Tuvblad C, Rijsdijk FV, Andershed H, Grann M, Lichtenstein P. A common genetic factor explains the association between psychopathic personality and antisocial behavior. Psychological medicine, 2007 37: p. 15–26. 17049102 10.1017/S003329170600907X

[21] Jones AP, Larsson H, Ronald A, Busfield P, McMillan A, Plomin R. et al Phenotypic and aetiological associations between psychopathic tendencies, autistic traits and emotion attribution. Criminal Justice and Behavior, 2009 36(11): p. 1198–1212.

[22] Haworth CMA, Davis OSP, Plomin R. Twins Early Development Study (TEDS): A Genetically Sensitive Investigation of Cognitive and Behavioral Development From Childhood to Young Adulthood. Twin Research and Human Development, 2012 p. 1–9. 10.1017/thg.2012.91 PMC3817931 23110994

[23] Oliver BR, Plomin R. Twins' Early Development Study (TEDS): A multivariate, longitudinal genetic investigation of language, cognition and behavior problems from childhood through adolescence. Twin Research and Human Genetics, 2007 10(1): p. 96–105. 17539369 10.1375/twin.10.1.96

[24] Goldsmith HH. A zygosity questionnaire for young twins: a research note. Behavior Genetics, 1991 21: p. 257–269. 1863259 10.1007/BF01065819

[25] Scott FJ, Baron-Cohen B, Bolton P, Brayne C. The CAST (Childhood Asperger Syndrome Test)—Preliminary development of a UK screen for mainstream primary-school-age children. Autism, 2002 6(1): p. 9–31. 11918111 10.1177/1362361302006001003

[26] Frick PJ. Antisocial Process Screening Device. Toronto: Multi Health Systems, 2001.

[27] Viding E, Blair RJ, Moffitt TE, Plomin R. Evidence for substantial genetic risk for psychopathy in 7-years-olds. Journal of Child Psychology and Psychiatry and Allied Disciplines, 2005 46: p. 592–597. 10.1111/j.1469-7610.2004.00393.x 15877765

[28] Goodman R. The strengths and difficulties questionnaire: A research note. Journal of Child Psychology and Psychiatry and Allied Disciplines, 1997 38(5): p. 581–586. 10.1111/j.1469-7610.1997.tb01545.x 9255702

[29] Ronald A, Happé F, Bolton P, Butcher LM, Price TS, Wheelwright S. et al Genetic heterogeneity between the three components of the autism spectrum: A twin study. Journal of the American Academy of Child and Adolescent Psychiatry, 2006 45(6): p. 691–699. 16721319 10.1097/01.chi.0000215325.13058.9d

[30] Robinson EB, Koenen KC, McCormick MC, Munir K, Hallett V, Happé F. et al A Multivariate Twin Study of Autistic Traits in 12-Year-Olds: Testing the Fractionable Autism Triad Hypothesis. Behavior Genetics, 2012 42(2): p. 245–255. 10.1007/s10519-011-9500-3 21927971 PMC3256271

[31] Ronald A, Larsson H, Anckarsäter H, Litchenstein P. A twin study of autism symptoms in Sweden. Molecular Psychiatry, 2011 16(10): p. 1039–1047. 10.1038/mp.2010.82 20644553

[32] Haworth CMA, Davis OSP, Plomin R. Twins Early Development Study (TEDS): A Genetically Sensitive Investigation of Cognitive and Behavioral Development From Childhood to Young Adulthood. Twin Res Hum Genet. 2013, 16(1). 10.1017/thg.2012.91 PMC3817931 23110994

[33] Neale MC, Cardon LR. Methodology for genetic studies of twins and families. Dordrecht: Kluwer Academic Publishers, 1992.

[34] Rijsdijk FV, Sham PC. Analytic approaches to twin data using structural equation models. Briefings in bioinformatics, 2002 3: p. 119–133. 12139432 10.1093/bib/3.2.119

[35] McGue M, Bouchard TJ. Adjustment of twin data for the effects of age and sex. Behavior genetics, 1984 14(4): p. 325–343. 6542356 10.1007/BF01080045

[36] Boker S, Neale M, Maes H, Wilde M, Spiegel M, Brick T. OpenMx: An Open Source Extended Structural Equation Modeling Framework. Psychometrika, 2011 76(2): p. 306–317. 23258944 10.1007/s11336-010-9200-6 PMC3525063

[37] R Development Core Team. R: A Language and Environment for Statistical Computing, 2011. p. {ISBN} 3-900051-07-0.

[38] Rijsdijk F. Independent Pathway Model, in Encyclopedia of Statistics in Behavioral Science. 2005, John Wiley & Sons, Ltd.

[39] Loehlin J. The Cholesky approach: A cautionary note. Behavior Genetics, 1996 26(1): p. 65-

[40] Ronald A, Hoekstra RA. Autism Spectrum Disorders and Autistic Traits: A Decade of New Twin Studies. American Journal of Medical Genetics Part B-Neuropsychiatric Genetics, 2011 156B(3): p. 255–274. 10.1002/ajmg.b.31159 21438136

[41] Bird G, Cook R. Mixed emotions: the contribution of alexithymia to the emotional symptoms of autism. Translational Psychiatry, 2013 3, e285; 10.1038/tp.2013.61 23880881 PMC3731793

[42] Scourfield J, Van den Bree M, Martin N, McGuffin P. Conduct problems in children and adolescents: a twin study. Archives of General Psychiatry, 2004 61(5): p. 489–96. 15123494 10.1001/archpsyc.61.5.489

[43] Viding E, Frick PJ, Plomin R. Aetiology of the relationship between callous-unemotional traits and conduct problems in childhood. British Journal of Psychiatry Supplement, 2007 49: s33–8. 10.1192/bjp.190.5.s33 17470941

[44] Rogers J, Viding E, Blair RJ, Frith U, Happé F. Autism spectrum disorder and psychopathy: shared cognitive underpinnings or double hit? Psychological Medicine, 2006 36(12): p. 1789–98. 17018169 10.1017/S0033291706008853

[45] Poythress NG, Douglas KS, Falkenbach D, Cruise K, Lee Z, Murrie DC, et al Internal consistency reliability of the self-report Antisocial Process Screening Device. Assessment. 2006, 13: p.107–113. 16443722 10.1177/1073191105284279

