# Supplementary material for: Examining the Genetic and Environmental Associations between Autistic Social and Communication Deficits and Psychopathic Callous-Unemotional Traits
Source: PLoS One. 2015 Sep 1;10(9):e0134331. doi: 10.1371/journal.pone.0134331 (PMC4556482; doi:10.1371/journal.pone.0134331)
Supplement: S1 Analysis — (DOCX) [file pone.0134331.s001.docx]

**Analysis S1: teacher-reported callous-unemotional traits and parent-reported social interaction and social communication difficulties.**

*Method:* Assessment of callous-unemotional traits by teacher-report in the sample indicated that the measure (described in the main text) had good internal consistency (Cronbach’s Alpha α = .75). The sample for teacher-rated callous-unemotional traits consisted of 5716 pairs (2030 monozygotic (MZ), 1889 dizygotic same-sex (DZ-SS) and 1797 dizygotic opposite-sex twin pairs (DZ-OS)). For social interaction and social communication subscales (derived from the Childhood Autism Spectrum Test) ([28](#_ENREF_28)), 6402 twin pairs were included (MZ N=2253; DZ SS N=2090; DZ OS N=2059) (as described in main text). Overall, there were 4667 pairs for whom ratings were available for callous-unemotional traits, as well as social interaction and social communication traits. Mean age of twins was 7.2 years (SD=.28) when rated by teachers on callous-unemotional traits. Exclusions described in the main text were applied before model fitting analysis. Analyses were run as described in the main text.

*Results:* Phenotypic correlations indicated modest associations between teacher-rated callous-unemotional traits and parent-rated social interaction (r=.13; p<.001), and teacher-rated callous-unemotional traits and parent-rated social communication (r=.18; p<.001). Cross-twin cross-trait correlations (Table S3) showed similar patterns to those reported for the analysis using parent report data, with monozygotic and dizygotic twin correlations showing a 2:1 ratio, suggesting that all three traits are significantly influenced by additive genetic influences. Cross-twin cross-trait correlations suggest that common genetic influences operating across the three dimensions may account for the small degree of phenotypic resemblance.

Results of the multivariate model-fitting analysis are shown in Table S4 and Figure S2. In the full independent pathway model, parameter estimates for several unique and common shared and non-shared environmental paths were equal to 0, suggesting a more parsimonious model might provide a better fit to the data. As described in the main analysis, we fitted 5 different nested sub-models, dropping model paths related to both unique and common shared and non-shared environmental parameters (Table S4). The best fitting model is shown in Figure S2, and from this we derive our estimates of the relative importance of independent and common etiological influences.

For teacher-reported callous-unemotional traits and parent-reported social interaction and social communication difficulties, most of the overall genetic influences on population variance were due to variable-specific genetic factors, explaining 85%, 74% and 61% of the heritability, respectively. The common genetic factor acting on all three traits explained a smaller proportion of the heritability: 15%, 26% and 39%, respectively. Trait-specific non-shared environmental influences were significant for only callous-unemotional and social interaction, whereas social communication appeared to be influenced by non-shared environmental influences that stemmed from the common non-shared environment path, although this common non-shared environment is effectively operating as trait-specific non-shared environment in the current model given that the common ‘E’ influences were not contributing to variance on social interaction of callous-unemotional.
