# Supplementary material for: Examining the Genetic and Environmental Associations between Autistic Social and Communication Deficits and Psychopathic Callous-Unemotional Traits
Source: PLoS One. 2015 Sep 1;10(9):e0134331. doi: 10.1371/journal.pone.0134331 (PMC4556482; doi:10.1371/journal.pone.0134331)
Supplement: S1 Fig — Abbreviations: A1-3 = Additive genetic factors modelled for each trait separately; C1&3 = Shared environmental factors modelled for each trait separately; E1-3 = Non-shared environmental factors modelled for each trait separately. Correlations for A, C and E factors between pairs of traits are shown. Paths from the A, C and E factors on to the traits reflect the estimated role of each of these factors in contributing to variance on the trait. Path C2 was removed as common environmental factors did not contribute significantly to variance on social interaction difficulties. (PDF) [file pone.0134331.s002.pdf]

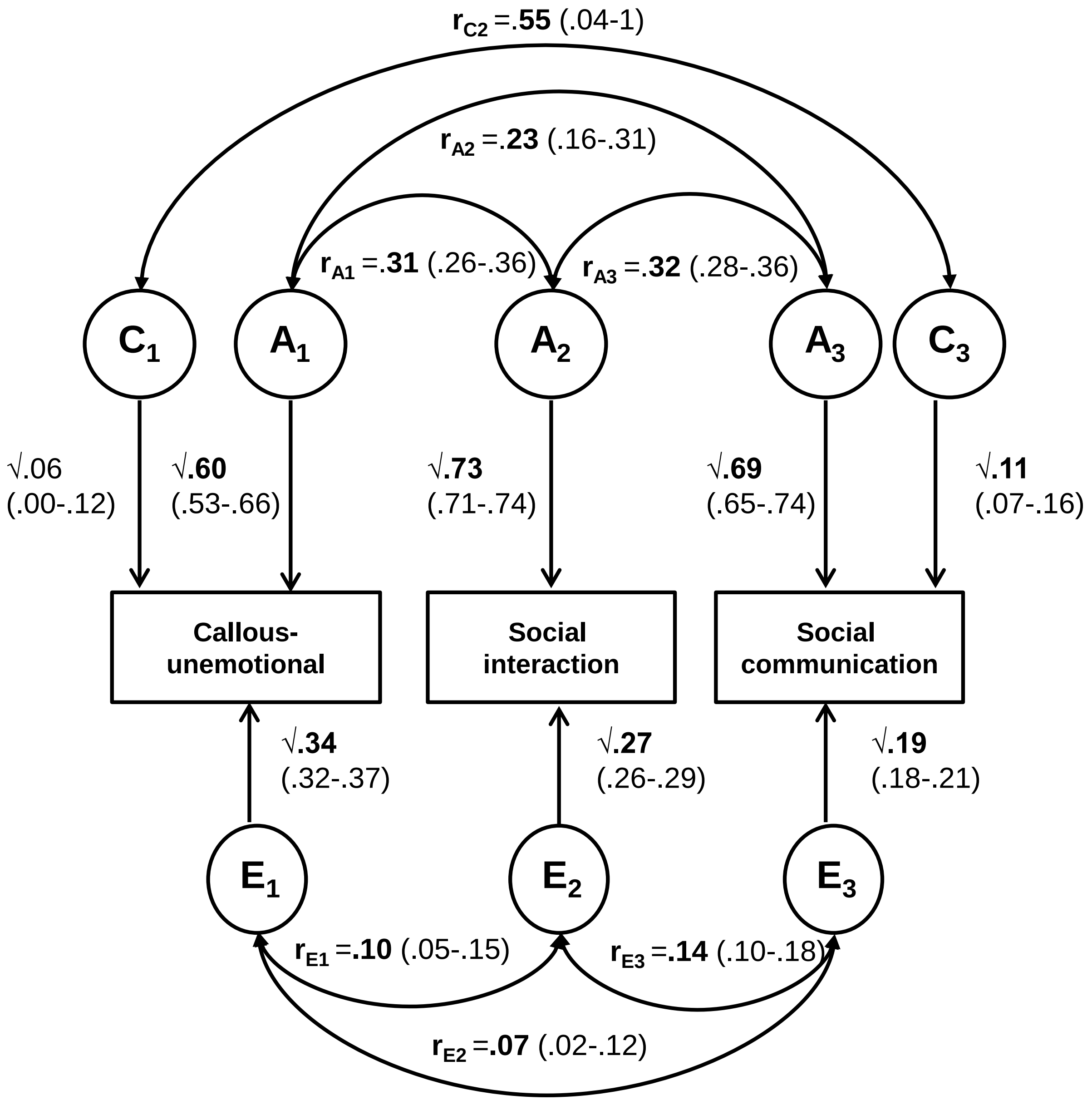

Supplemental Figure 1: Cholesky ACE model: Correlated Factors Solution outputs using parent-report data on callous-unemotional traits, social interaction and social communication. Abbreviations: $A_{1\text{-}3}$ = Additive genetic factors modelled for each trait separately; $C_{1\ \&\ 3}$ = Shared environmental factors modelled for each trait separately; $E_{1\text{-}3}$ = Non-shared environmental factors modelled for each trait separately. Correlations for A, C and E factors between pairs of traits are shown. Paths from the A, C and E factors onto the traits reflect the estimated role of each of these factors in contributing to variance on the trait. Path C_2 was removed as common environmental factors did not contribute significantly to variance on social interaction difficulties
