# Supplementary material for: Examining the Genetic and Environmental Associations between Autistic Social and Communication Deficits and Psychopathic Callous-Unemotional Traits
Source: PLoS One. 2015 Sep 1;10(9):e0134331. doi: 10.1371/journal.pone.0134331 (PMC4556482; doi:10.1371/journal.pone.0134331)
Supplement: S2 Fig — Paths estimates are expressed as the proportion of variance accounted for by common or specific aetiological factors. For callous-unemotional, 15% of overall genetic influences are due to shared factors and 85% due to trait specific factors. For social interaction, 26% of overall genetic influences are due to shared factors and 74% due to trait specific factors. For social communication, 39% of overall genetic influences are due to shared factors and 61% due to trait specific factors. Dotted arrows indicate a non-significant path estimate. Ac = common genetic factors; Ec = common non shared environmental factors; As = specific genetic factors; Cs = specific shared environmental factors; Es = specific non shared environmental factors. h2 = proportion of variance on the trait explained by additive genetic influences; c2 = proportion of variance explained by shared environmental influences; e2 = proportion of variance explained by non-shared environmental influences. (PDF) [file pone.0134331.s003.pdf]

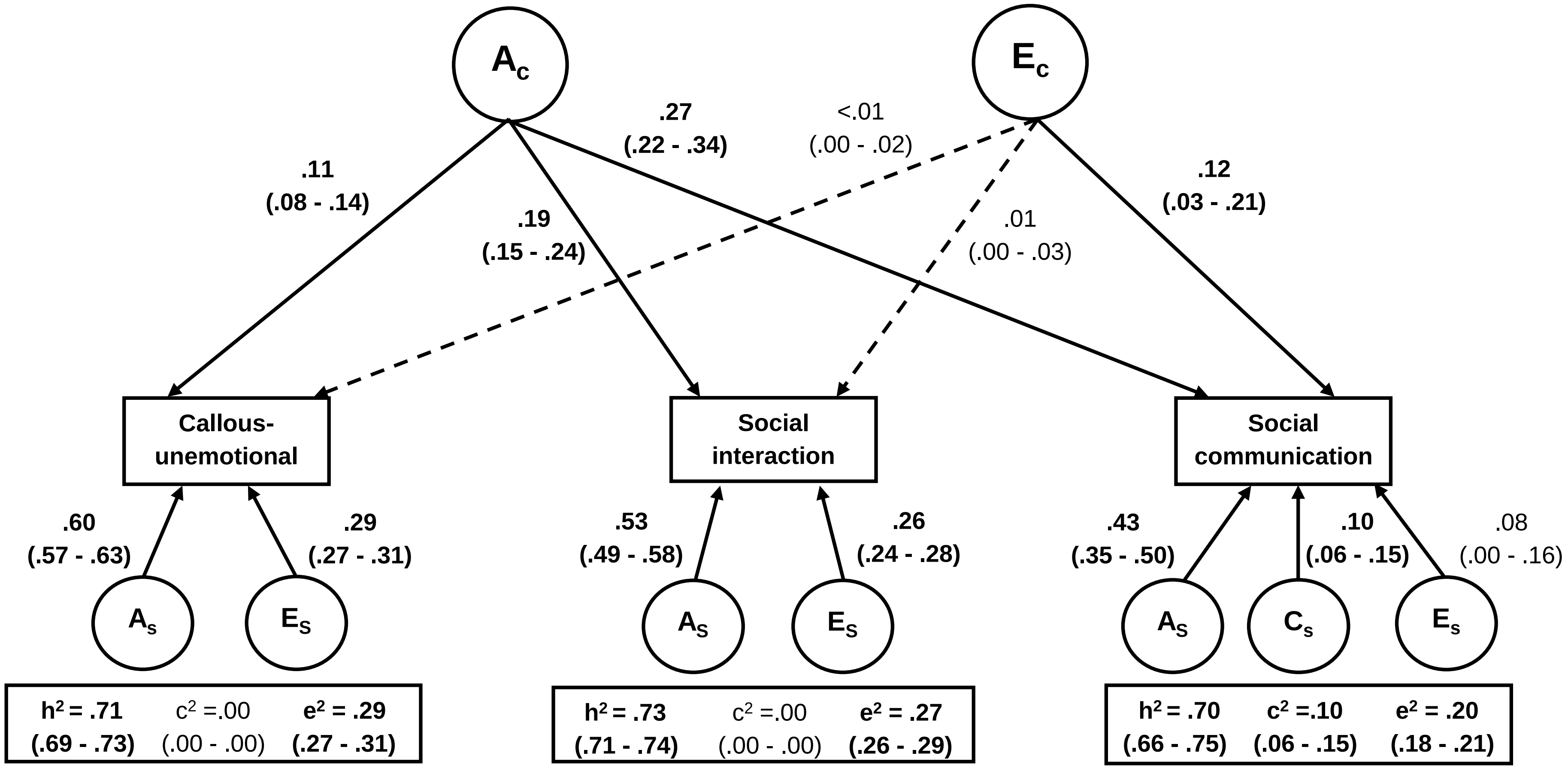


**Supplemental Figure 2:** Path diagram of the reduced Independent Pathway model. Paths estimates are expressed as the proportion of variance accounted for by common or specific etiological factors. For callous-unemotional, 15% of overall genetic influences are due to shared factors and 85% due to trait specific factors. For social interaction, 26% of overall genetic influences are due to shared factors and 74% due to trait specific factors. For social communication, 39% of overall genetic influences are due to shared factors and 61% due to trait specific factors. Dotted arrows indicate a non-significant path estimate. $A_c$ = common genetic factors; $E_c$ = common non shared environmental factors; $A_s$ = specific genetic factors; $C_s$ = specific shared environmental factors; $E_s$ = specific non shared environmental factors. $h^2$= proportion of variance on the trait explained by additive genetic influences; $c^2$= proportion of variance explained by shared environmental influences; $e^2$ = proportion of variance explained by non-shared environmental influences.
