# Supplementary material for: Examining the Genetic and Environmental Associations between Autistic Social and Communication Deficits and Psychopathic Callous-Unemotional Traits
Source: PLoS One. 2015 Sep 1;10(9):e0134331. doi: 10.1371/journal.pone.0134331 (PMC4556482; doi:10.1371/journal.pone.0134331)
Supplement: S1 Table — (DOCX) [file pone.0134331.s004.docx]

**Table S1: Items measuring callous-unemotional, social interaction and social communication traits.**

| **Callous-unemotional^a^** |
| --- |
| 1) Does not show feelings or emotions |
| 2) Helpful if someone is hurt, upset or feeling ill |
| 3) Feels bad or guilty when s/he does something wrong |
| 4) Has at least one good friend |
| 5) Considerate of other people’s feelings |
| 6) Kind to younger children |
| 7) Is concerned how well s/he does at school |
| 8) His/her emotions seem shallow and not genuine |
| **Social interaction^b^** |
| 1) Does s/he join in playing games with other children easily? |
| 2) Does s/he come up to you spontaneously for a chat? |
| 3) Is it important to him/her to fit in with the peer group? |
| 4) When s/he was 3 years old, did s/he spend a lot of time pretending (e.g. play-acting being a superhero, or holding teddy’s tea parties)? |
| 5) Does s/he have friends, rather than just acquaintances? |
| 6) Does s/he often bring you things s/he is interested in to show you? |
| 7) Are people important to him/her? |
| 8) Does s/he play imaginatively with other children, and engage in role-play? |
| 9) Does s/he make normal eye contact? |
| 10) Is his/her social behaviour very one-sided and always on his/her terms? |
| 11) Does s/he prefer imaginative activities such as play-acting or story-telling, rather than numbers or lists of facts? |
| 12) Does s/he care how s/he is perceived by the rest of the group? |
| **Social communication^c^** |
| 1) Does s/he find it easy to interact with other children? |
| 2) Can s/he keep a two-way conversation going? |
| 3) Does s/he enjoy joking around? |
| 4) Is s/he good at turn-taking in conversation? |
| 5) Does s/he tend to take things literally? |
| 6) Does s/he have difficulty understanding the rules for polite behaviour? |
| 7) Is his/her voice unusual (e.g. overly adult, flat or monotonous)? |
| 8) Does s/he often do or say things that are tactless or socially inappropriate? |
| 9) Does s/he sometimes say ‘you’ or ‘s/he’ when s/he means I? |
| 10) Does s/he sometimes lose the listener because of not explaining what s/he is talking about? |
| 11) Does s/he often turn conversations to his/her favourite subject rather than following what the other person wants to talk about? |
| 12) Does s/he have odd or unusual phrases? |

^a^For callous-unemotional traits, items 2-7 were reverse scored. ^b^For social interaction, all items, apart from no. 10, were reverse scored. ^c^For social communication traits, items 1-7 were reverse scored.
