# Supplementary material for: Examining the Genetic and Environmental Associations between Autistic Social and Communication Deficits and Psychopathic Callous-Unemotional Traits
Source: PLoS One. 2015 Sep 1;10(9):e0134331. doi: 10.1371/journal.pone.0134331 (PMC4556482; doi:10.1371/journal.pone.0134331)
Supplement: S3 Table — (DOCX) [file pone.0134331.s006.docx]

**Table S3: Cross-twin within-trait and cross-trait correlations, stratified by zygosity and gender using teacher-report data for callous-unemotional traits, and parent-report data for social interaction and social communication difficulties.**

| **Monozygotic males** | | | | |
| --- | --- | --- | --- | --- |
|  | | ***Twin 1*** | | |
|  |  | **CU** | **SI** | **SC** |
| ***Twin 2*** | **CU** | **.72 (.69 - .75)** | - | - |
|  | **SI** | .18 (.14 - .21) | **.76 (.74 - .78)** | - |
|  | **SC** | .20 (.17 - .23) | .26 (.23 - .28) | **.81 (.79 - .82)** |
| **Dizygotic males** | | | | |
|  | | **Twin 1** | | |
|  |  | **CU** | **SI** | **SC** |
| ***Twin 2*** | **CU** | **.30 (.24 - .35)** | - | - |
|  | **SI** | .00 (-.04 - .05) | **.31 (.25 - .36)** | - |
|  | **SC** | .08 (.04 - .13) | .05 (.01 - .10) | **.40 (.35 - .45)** |
| **Monozygotic females** | | | | |
|  | | **Twin 1** | | |
|  |  | **CU** | **SI** | **SC** |
| ***Twin 2*** | **CU** | **.69 (.66 - .72)** | - | - |
|  | **SI** | .11 (.07 - .14) | **.71 (.68 - .73)** | - |
|  | **SC** | .14 (.11 - .18) | .20 (.17 - .22) | **.80 (.79 - .82)** |
| **Dizygotic females** | | | | |
|  | | **Twin 1** | | |
|  |  | **CU** | **SI** | **SC** |
| ***Twin 2*** | **CU** | **.43 (.37 - .47)** | - | - |
|  | **SI** | .03 (-.02 - .07) | **.35 (.30 - .40)** | - |
|  | **SC** | .08 (.03 - .12) | .11 (.08 - .15) | **.50 (.46 - .54)** |
| **Dizygotic opposite sex** | | | | |
|  | | **Twin 1** | | |
|  |  | **CU** | **SI** | **SC** |
| ***Twin 2*** | **CU** | **.33 (.29 - .37)** | - | - |
|  | **SI** | .04 (.01 - .08) | **.28 (.24 - .32)** | - |
|  | **SC** | .10 (.07 - .14) | .11 (.08 - .14) | **.46 (.42 - .49)** |

Abbreviations: CU = Callous-unemotional; SI = Social interaction, SC= Social Communication.
