# Supplementary material for: Examining the Genetic and Environmental Associations between Autistic Social and Communication Deficits and Psychopathic Callous-Unemotional Traits
Source: PLoS One. 2015 Sep 1;10(9):e0134331. doi: 10.1371/journal.pone.0134331 (PMC4556482; doi:10.1371/journal.pone.0134331)
Supplement: S4 Table — (DOCX) [file pone.0134331.s007.docx]

**Table S4: Model fitting results for the Independent Pathway Models using teacher-report data for callous-unemotional traits, and parent-report data for social interaction and social communication.**

| **Model** | **Number of parameters** | **Δ parameters (relative to full model)** | **-2*log-likelihood** | **Δ likelihood – χ^2^ value (relative to full model)** | **p-value** |
| --- | --- | --- | --- | --- | --- |
| IP full ACE | 21 | - | 97692.72 | - | - |
| M1^a^ | 18 | 3 | 97695.35 | 2.62 | .45 |
| M2^b^ | 15 | 6 | 97715.60 | 22.87 | .001 |
| M3^c^ | 16 | 5 | 97695.35 | 2.62 | .76 |
| M4^d^ | 14 | 7 | 97759.59 | 67.16 | .001 |
| M5^d^ | 15 | 6 | 97710.73 | 18.00 | .006 |

Abbreviations: IP full ACE = an Independent Pathway ACE model containing path estimates for additive genetic, shared environmental and non-shared environmental influences acting on each trait separately (specific path estimates), and acting on all three traits (common path estimates, quantifying overlapping etiological influences).

^a^ In model M1, common shared environment paths were dropped. Model M1 provided a better fit to the data than the full IPM.

^b^ In model M2, common and unique shared environment paths were dropped. Model M2 fitted worse compared to the full IPM.

^c^ To define more closely the potential source of significant unique shared environmental influences acting on either of the variables explaining the drop in fit for M2, sub-model M3, in which common and specific shared-environment paths for callous-unemotional and social interaction only were dropped. M3 was the best fitting model (χ^2^=2.62, p=0.76).

^d^ The remaining 2 sub-models, M4 and M5, were fitted to test whether dropping common non-shared environment paths for callous-unemotional and social interaction (M4); or dropping common non-shared environment only for callous-unemotional (M5) improved model fit. However, both models fitted worse, so these paths could not be taken out.
